# Overgrowth of Douglas fir (*Pseudotsuga menziesii* Franco) stumps with regenerative tissue as an example of cell ordering and tissue reorganization

**DOI:** 10.1007/s00425-014-2142-y

**Published:** 2014-08-13

**Authors:** Urszula Zajączkowska

**Affiliations:** Department of Forest Botany, Warsaw University of Life Sciences (WULS), 159 Nowoursynowska Str, 02-776 Warsaw, Poland

**Keywords:** Cell polarity, Circular pattern, Digital image analysis, Morphogenetic field, Xylogenesis

## Abstract

**Stump overgrowth may serve as a unique model for studying cellular reorganization and mechanisms responsible for cell polarity changes during the process of vascular tissue differentiation from initially unorganized parenchymatous cells.**

Cellular ordering and tissue reorganization during the overgrowth process of the transverse surfaces of Douglas fir stumps in forest stand was studied. At the beginning of stump overgrowth, the produced parenchymatous cells form an unorganized tissue. Particular parenchyma cells start arranging into more ordered structures which resemble rays. Application of digital image analysis software based on structure tensor was used. The analysis showed that at this stage of tissue development, cellular elements display a wide range of angular orientation values and attain very low coherency coefficients. The progress of the tissue differentiation process is associated with the formation of local regions with tracheids oriented circularly around the rays. This coincides with an increase in the range of angular orientations and greater values of coherency coefficients. At the most advanced stage of tissue development, with tracheids arranged parallelly in longitudinal strands, the degree of cell ordering is the highest what is manifested by the greatest values attained by coherency coefficients, and the narrow range of angular orientations. It is suggested that the ray-like structures could act as organizing centers in the morphogenetic field responsible for differentiation of the overgrowth tissue. The circular pattern of tracheids around rays in the initial phase of tissue development can be interpreted in terms of local rotation of the morphogenetic field which afterward is transformed into irrotational field. This transformation is noted by the presence of tracheids arranged parallelly in longitudinal strands. The possible involvement of a mechanism controlling cell polarity with respect to auxin transport is discussed.

## Introduction

Plant morphogenesis includes all the processes involved in the development of an organism into its eventual form and structure (Wardlaw 1952). These activities include the formation of organs and the differentiation of tissues. Explaining the mechanisms responsible for the coordinated growth and differentiation at the various levels of organization, and enabling the development of an integrated form and structure characteristic for a given organism, are presently main aims of biological studies. Comprehensive investigations in this field involve various morphological, anatomical, cytological, physiological, molecular, and biophysical techniques as well as mathematical modeling (Gamborg et al. 1973; Vasil and Vasil 1981; Murata and Orton 1987; Sujatha and Mukta 1996). Experiments in the field of plant morphogenesis often use the processes of wound healing and regeneration in tissues and organs as model systems. Standard methods that are frequently used include analyses of cytodifferentiation and organogenesis in callus tissues, wounded organs, or tissues isolated in sterile cultures.

New research perspectives in plant morphogenesis seem to be provided by the previously practically unused model of the “living stumps” (Lanner 1961; Börmann 1966; Liphschitz et al. 1987; Lev-Yadun 2011). Such stumps are overgrown with regenerative tissues after tree cutting. Overgrowth of the transverse surfaces is a long-term process that can continue for many years after tree-stem cutting. This phenomenon, although described a long time ago for *Abies alba*, *Larix decidua, Pinus sylvestris*, and *Pseudotsuga* *menziessi* (Kobendza 1955; Tumiłowicz 1958), has not been subjected to further detailed studies. According to the early workers, tissue covering the stump surface shows structures similar to annual wood rings. As suggested, such structures could be formed in stumps with root systems grafted to intact trees, surrounding the stumps in forest stand and supplying them with nutrients.

However, it is known that in the growth processes of trees, cambium plays a particularly important role (Romberger et al. 1993; Larson 1994; Lachaud et al. 1999). As cambial cells divide, their xylem derivatives, arranged in radial rows, form the tissue of annual wood rings. It is also well known that tree growth is strongly affected by the polar transport of auxin from apical stem sources, inducing periclinal divisions in the cambium (Zajączkowski et al. 1984; Sachs 1984, 1991; Aloni 1989; Sablowski 2007). According to the commonly accepted theory, auxin signal from shoot apical meristems and actively growing leaves is transported basipetally in the stem and acropetally in the roots, toward their apices (Zimmermann and Brown 1971; Romberger et al. 1993; Aloni 2013). Thus, if stumps were supplied by a source of auxin located in the apical stem meristems and leaves of the surrounding intact trees via their root systems grafted with stump roots, the pathway of auxin signal transduction should proceed basipetally in stems and acropetally in roots of intact trees to afterwards sharply change into basipetal in the stump roots and acropetal in the stump itself. Such a phenomenon, requiring reverse polarity in auxin polar transport over a long distance in the stump of the cut tree and its root system, has not yet been described.

Authors presenting the cellular structure of overgrowth tissue in stumps reported differences at various regions of the regenerative tissue. The occurence of irregularly arranged parenchymatous cells and development of tracheidal cells of irregular shapes as well as arranged in approximately regular radial files and forming annual rings of wood were described (Kobendza 1955; Tumilowicz 1958). However, this issue has not been examined in detail. The main aim of the present study was to investigate the role of positional control in the induction of cell differentiation and reorganization of the cell arrangement in overgrowth tissues of Douglas fir stumps. Changes in the level of orientation and in the arrangement of cell structures proceeded during the development of the overgrowth tissue were assessed quantitatively by digital image analysis (Fonck et al. 2009; Rezakhaniha et al. 2011; Zajączkowska 2014).

## Materials and methods

**Fig. 1:**
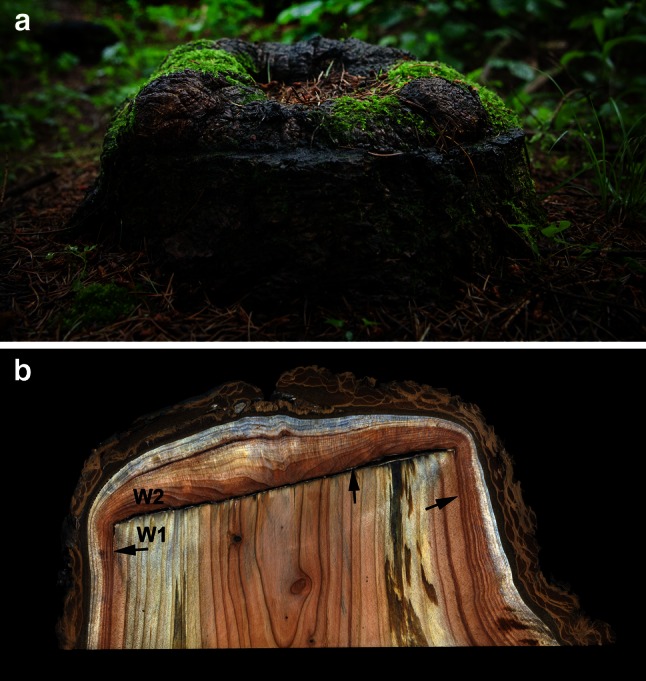
Overgrown stump of Douglas fir formed during a 30-year period, after tree cutting. **a** Tree stump in the forest stand covered with bark tissues. **b** Longitudinal section of the stump with annual rings of wood (W) formed before (*1*) and after (*2*) cutting; *arrows*—boundary between wood formed before and after stem cutting

The study was performed on three stumps of Douglas fir (*Pseudotsuga menziesii* Franco) overgrown with regenerative tissues. The stumps were located in a forest stand of the Warsaw University of Life Sciences (WULS) Arboretum, in Rogów (Central Poland). Trees were cut using the thinning procedures applied in the Douglas fir forest stand. After the trees had been cut, the stumps (about 20 cm high) remained in the area for ca. 30 years. During this time, the surface of the stumps (ca. 25 cm in diameter) became overgrown with up to 4 cm of thick tissue. The outer layer of the overgrowth tissue was covered with a layer of cork (Fig. 1a). Transverse, radial, and tangential microscopic sections of the overgrowth tissue were cut on sliding microtome. Microscopic examinations were carried out on sections stained with phloroglucinol–HCl reagent to indicate the presence of lignin. The samples for the maceration of stump wood and overgrown tissues were incubated at 56 °C for 6 days in a solution of glacial acetic acid: water: hydrogen peroxide (30 %) (5:4:1, by vol.). The separated cells were stained with safranine. An Olympus optical microscope integrated with the Cell^P software was used for anatomical investigations.

An analysis of the cell ordering was performed by measuring local cell orientation on tangential sections of the overgrowth tissue. Measurements were carried out using Fiji software (Schindelin et al. 2012), and the OrientationJ plugin digital image processing tool based on structure tensor (Fonck et al. 2009; Rezakhaniha et al. 2011). Structure tensors are frequently used in image analysis processing (Jahne 1993; Bigun et al. 2004). The tensor is estimated for each pixel of an image and spatial derivatives in principal directions *x* and *y* are computed using cubicB-spline interpolation (Unser et al. 1993). The method can estimate the dominant directions in the environment of the pixel gradient and the coherency coefficients in the selected regions of image. The software outputs a color-coded map showing the angles of oriented structures in the image. The coherency coefficient may vary between 1 and 0. Values close to 1, geometrically depicted as a slim ellipse, represent regions with a highly oriented structure. Values close to 0, pictured as a circle, indicate no preferential orientation. The two coherency coefficient values refer to anisotropic and isotropic regions, respectively.

## Results

**Fig. 2:**
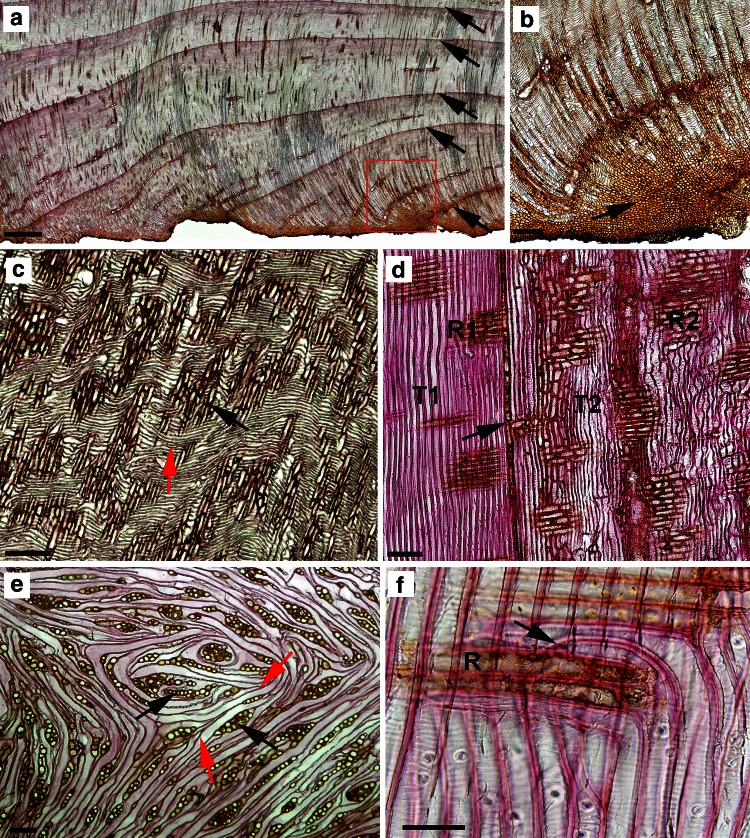
Microscopic sections of the overgrown tissue. **a** Transverse section of regenerative tissue with distinctive layers of annual rings of wood. **b** Tissue sector marked on a under higher microscopic magnification; the callus-like parenchymal zone with unorganized cells formed at the beginning of tissue regeneration (*arrow*). **c** Radial and **e** tangential sections of overgrown tissues in the region of the circular tracheary elements’ differentiation (*red arrow*) around the ray-like structures (*black arrows*). **d** Radial section of stump tissue, below the cut surface, formed before and after stem cutting. *T* and *R*, tracheids and rays formed before (*1*) and (*2*) after cutting; *arrow*, boundary between xylem formed before and after stem cutting. **f** Radial section of stump xylem below the cut surface, formed after stem cutting, under higher microscopic magnification. R, xylem ray; *arrow*, tip of tracheid. *Scale bars* = 350 μm (**a**), 50 μm (**b**), 150 μm (**c**, **d**), 100 μm (**e**), 60 μm (**f**)

The outer layers of overgrowth tissue were similar to cork tissues (Fig. 1a). Below this region of bark were layers of wood tissue arranged in annual rings (Fig. 1b). Microscopic studies of overgrowth tissue close to the sampled cut surface of the stump showed the presence of irregularly arranged parenchymatous callus-like cells (Fig. 2b). As the distance from the cut surface increased, there was an enhancement in the ordering of parenchyma cells. At greater distances, some of the cells were organized in forms differentiated into structures resembling secondary xylem rays (Fig. 2a). Between these structures, initially the parenchyma cells and subsequently tracheid-like cells were recorded. Tracheids formed structures resembling annual rings of wood with clearly marked boundaries (Fig. 2b). Closest to the cut surface, in the newly formed zone of xylem, the rays were arranged obliquely with respect to the boundary of annual increments. At farther distances, the xylem rays became perpendicularly oriented with respect to annual ring boundaries. Microscopic inspection of radial sections from the mid-part of the stump overgrowth tissues revealed presence of the ray-like structures and a specific wavy pattern of the axial tracheary elements (Fig. 2c).

A circular pattern of the tracheary elements around the rays was manifested when the tangential sections from a similar region of the overgrowth tissue were observed (Fig. 2e). It is worth noting that the tracheids in this region were distinguished by sharply ended tips located two adjacent tracheids and that such situation is commonly observed in tissues characterized by intrusive cell growth. Modifications in the xylem structure also occurred in the stump wood formed below the cut surface (Fig. 2d). As it is seen on radial sections, the xylem rays are oriented perpendicularly in the annual rings of wood formed both before and after stem cutting. The number of rays and the size of the xylem ray cells in the newly formed wood, though, were greater. Along the length of newly differentiated axial tracheids, a slight wavy-like pattern could be distinguished. In some of the tracheids, a sudden change in cell orientation was observed: part of the cell was oriented parallel and the other part of the same cell was perpendicular to the stump axis (Fig. 2f).

**Fig. 3:**
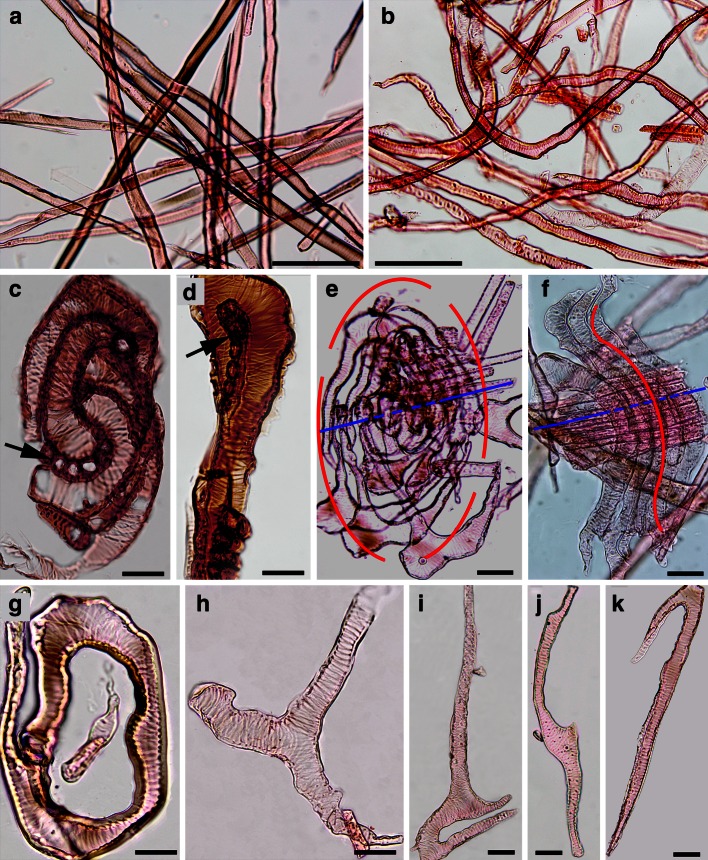
Structure of elements of Douglas fir wood formed in stump before the tree was cut (**a**) and in the stump’s overgrown tissues (**b**−**k**). **a** Macerated axial tracheids in normal wood of straight shape. **b** Macerated tracheary elements of the overgrown, different shapes. **c** Short tracheary elements formed a spiral-like arrangements around parenchymatous cells (*arrow*) located in the tracheid concave region. **d** Bent end of tracheary element with the parenchyma cells (*arrow*) located in the concave side. **e** Circular (*red line*) arrangement of tracheary elements around xylem ray-like parenchyma cells (*blue line*). **f** Rounded shape of tracheary elements (*red line*) near the xylem ray-like structure. **g**−**h** Examples of various shape of tracheary elements in overgrown tissue: circular **g**, furcated **h**, bent with local concave (*arrow*) region (i), with two very short lateral tips (*arrow*) in central cell region **j**, with curved end **k**. *Scale bars* = 200 μm (**a**, **b**), 50 μm (**c**, **g**−**k**), 40 μm (**d**), 100 μm (**e**, **f**)

Microscopic observation of the macerated tissues revealed various forms of tracheary elements in the overgrown tissues, whereas the axial tracheids formed before the tree was cut were relatively uniform and showed the straight shape typical for conifer wood (Fig. 3a, b). In the overgrown tissue near the cut stump’s surface, the tracheary elements were relatively short and formed arrangements resembling a whirled pattern in which isodiametric parenchymatous cells were also present (Fig. 3c). Some of the tracheary elements were bent near the tip and on the concave bent side, the parenchyma cells were visible (Fig. 3d). When there was an aggregated group of cells isolated from the tissue, a circular arrangement of tracheary elements around ray-like structures was noticed (Fig. 3e). In some cases, the tracheary elements were rounded near the rays, and at farther distances were straighter (Fig. 3f). Microscopic inspection of individual cells separated from the stump overgrown tissues, revealed variously shaped tracheary elements e.g., circular (Fig. 3g), ramified (Fig. 3h), locally folded with a concave region (Fig. 3i), having two additional small lateral tips in the central region of the cell (Fig. 3j), and curved near the cell tip (Fig. 3k). The spiral thickening of the cell wall, typical for axial tracheids of Douglas fir, was also observed in the tracheary elements of the overgrown tissue.

**Fig. 4:**
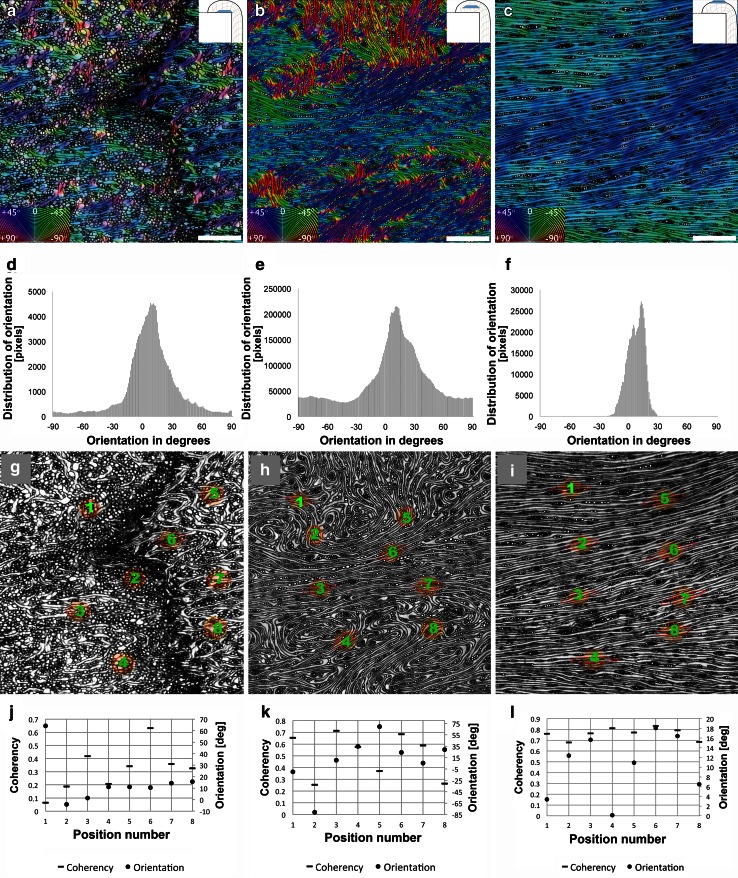
Cellular organization of the regenerative tissues of a Douglas fir stump’s overgrowth. Various locations with respect to the cut surface of the stump are shown. **a**, **d**, **g**, **j** Tissue zone close to the cut surface (1–3 mm). **b**, **e**, **h**, **k** Central region of overgrowth tissue (16th annual increment). **c**, **f**, **i**, **l** External part of overgrowth tissue (28th annual increment). **a**, **b**, **c** Color-coded maps of local predominant orientations obtained by digital image processing software. The maps show the angle orientations (*degrees*) in the transverse sections of tissue regions seen on the microscopic images. **d**, **e**, **f** Histogram distribution of predominant angle orientations in tissue regions on color-coded maps shown in **a**, **b**, and **c**, respectively. **g**, **h**, **i** Coherency coefficients represented geometrically as ellipses on sample positions located on the microscopic images of the same tissue regions seen in **a**, **b** and **c**, respectively. **j**, **k**, **l** Numerical values of angle orientations (*degrees*) and coherency coefficients for six sample positions on each of the tissue region shown in **g**, **h**, and **i**, respectively. *Scale bars* = 500 μm

Cell ordering within the overgrowth tissue was estimated using the OrientationJ image analysis plugin based on structure tensor. The survey was conducted on a representative group of microscopic images having a total of 18 tangential sections cut in overgrowth tissue at three zones of various distances from the stump’s cut surface: (a) close to the cut surface (ca. 1−3 mm), (b) central region of the overgrowth tissue (ca. 15−18 annual increments), and (c) the external part of the overgrowth tissue (27−29 annual increments). In each zone, five tangential sections were measured. Exemplary results for each zone are shown in Fig. 4. Results presented on color-coded maps illustrate the predominant cell orientation in tissue images. Measurements near the cut surface of the stump (Fig. 4a, d, g, j) revealed that predominant orientations ranged between −30° and +60°, with a maximum density at an angle of ca. +15°, with respect to the vertical image axis (Fig. 4a, d). More detailed analyses carried out for this region, including groups of interlacing suberized parenchyma cells and tracheid-like cells, showed relatively low values of coherency coefficients (Fig. 4g, j). The central zone of the overgrowth tissue (Fig. 4b, e, h, k), with irregularly arranged tracheids and xylem rays, had a predominant orientation of between −90° and +90° with a maximum density at ca. +15° (Fig. 4b, e). In this region, coherency coefficients were higher as compared to the zone close to the cut surface of the stump (Fig. 4h, k). In images taken from the external tissue region (Fig. 4c, f, i, l), the tracheids and xylem rays were regularly distributed and displayed a narrow range of predominant cell orientation, varying between −20° and +30° with a maximum density at an angle of ca. +10° (Fig. 4c, f). This region was also typified by the highest values of coherency coefficients (Fig. 4i, l). The increase of the coherency coefficients estimated for the three successively formed regions of the overgrown stump tissues were statistically significant. The mean and standard deviation values for the zone near the stump’s surface, central, and external regions were 0.317 ± 0.171, 0.512 ± 0.189, and 0.762 ± 0.055, respectively.

## Discussion

The present studies showed that at the beginning of stump overgrowth the produced parenchymatous cells form an unorganized, irregular tissue, however, including particular parenchyma cells starting to arrange into more ordered structures resembling rays. Tracheary elements appearing in the tissue usually differentiated around these primary rays and formed circular structures. This may indicate that such primary rays are a tissue constituent necessary for the development of vascular cells, which divide and differentiate into tracheary elements. The circular shape of tracheid-like cells, which were differentiated around the rays, may suggest that these cells can be considered as organizing centers responsible for the development of cambial-like cells in the morphogenetic field.

At the beginning of stump–tissue development, the spatial arrangement of ray parenchyma cells and tracheids showed a low degree of ordering. Application of digital image analysis software showed that at this stage of tissue development, cellular elements displayed a wide range of angular orientation values and attained very low coherency coefficients. Progress of the tissue differentiation process was associated with formation of local regions with tracheids circularly oriented around the rays. This coincides with an increase in the range of angular orientation and greater values of coherency coefficients, which may indicate a higher degree of cell ordering. At the most advanced stage of tissue development with tracheids arranged in parallel longitudinal strands, the degree of cell ordering is the highest. The greatest values attained by the coherency coefficients, and the narrow range of angular orientations show that there is a high degree of ordering. Thus, results of the present study indicate that digital image analysis tools based on structure tensors appear to provide a very helpful and fast method for measuring cellular organization in various regions of regenerating plant tissue.

The example of overgrowth in Douglas fir tree stumps after cutting suggests that “living stumps” could serve as one of the most interesting and unique models for studying the mechanisms of plant morphogenesis. As it is known, one of fundamental issues in morphogenesis is to explain how local interactions between elementary components lead to the collective behavior and development of highly organized biological systems (Klebe et al. 1991; Volfson et al. 2008). In bacterial and animal systems, this can be achieved by ordering and differentiation of mobile cells. In the case of plant tissues comprised of immobile cells, the mechanism of cell reorientation must involve unequal symplastic growth as well as anticlinal divisions, meristematic cell elimination, and intrusive growth. The last three morphogenetic events are known to be responsible for cell orientation changes that in tree stems may result in a domain pattern of cambium (Hejnowicz and Krawczyszyn 1969; Krawczyszyn 1973; Pyszyński 1972; Zagórska-Marek 1984) or may result in grain and interlocked wood (Hejnowicz 1971; Krawczyszyn 1972; Harris 1989). Noticeable and fast changes in the spatial orientation of xylem cells were also described for various types of cambium damage, resulting e.g., from cutting of phloem bridges (Kirschner et al. 1971) or spiral incisions, in several coniferous trees (Harris 1969, 1973; Savidge and Farrar 1984; Zagórska-Marek and Little 1986). Most authors link the observed changes to anticlinal divisions of cambium proceeding in the preferred direction, and to intrusive growth. Occurrence of circular vessels and tracheids, representing special types of the whirled grain pattern, seems particularly interesting. Such observations were previously reported for plants mainly affected by mechanically induced wounds (Aloni and Wolf 1984; Lev-Yadun and Aloni 1991), insect-caused wounds (Liphschitz and Mendel 1987), stem grafts (Küster 1899), root grafts (Neef 1922), and tumors (Włoch 1976). Studies by Sachs and Cohen (1982), who induced the formation of circular vessels above transverse wounds in *Pisum sativum* and *Phaseolus vulgaris*, are of particular interest. According to these authors, tissue disruption resulted in the reversal of the original basipetal auxin transport. The inductive signals that were produced promoted a circular pattern in cambial cells. Induction of circular vessels by exogenously applied auxin was also observed in *Fraxinus excelsior* for isolated stem segments (Kurczyńska and Hejnowicz 1991). Such structures were recorded in intact plants as well. For example, Hejnowicz and Kurczyńska (1987) described circular vessels present above axillary buds in eight angiosperm tree species. The authors suggested that these vessels occurred in vortices resulting from circular polarity in the cambial region above the bud. Interesting information on the issue was also provided by Lev-Yadun and Aloni (1990), who found circular structures in bifurcation zones of two or more branches, in the contact zone between two opposite vascular patterns, in a number of coniferous and dicotyledonous species. The authors explained their observation with a modified hypothesis on polar auxin transport, following Sachs and Cohen (1982).

There is, however, a difference between circular patterns of tracheary elements described in the above literature and those reported in the present study. Previously, such structures were found in isolated trunk or root regions exhibiting axial polarity typical of trees. The circular pattern resulted from the local reorganization of this typical polar structure. In overgrowth tissues of Douglas fir stumps, circular tracheary structures appeared to be formed at the early stages of tissue differentiation, followed by the development of polarity. Seemingly, these circular elements are the result of an intrusive growth of cells around the primary rays. Following the suggestion of Hejnowicz and Kurczyńska (1987), we may regard the circular tracheary structures in intact tree stems to be formed in local vortices resulting from circular polarity in the highly anisotropic (axially polar) cambial region. In the case of overgrown stumps, such cell polarity-associated vortices should represent the first stage of organization in the initially irregular isotropic tissue, eventually developing into highly ordered axially polar structures.

With the mathematical field theory (Korn and Korn 1968) in mind, one may speculate about the hypothetical morphogenetic field involved in the circular pattern. Since the field is polar, it should be considered as a vector field F(r). In a three-dimensional rectangular Cartesian system with coordinates (*x*, *y*, *z*), the position vector *r* ≡ *x*i + *y*j + *z*k, where i, j, k are the unit vectors in their respective directions. Such a vector field throughout a specified *V* region can occur as an irrotational vector field or can exhibit vector rotation if, for every point of *V*, curl F(*r*) = 0 and curl F(*r*) ≠ 0, respectively.

Curl F (*r*) ≡ ∇ × F is a vector operator that describes the infinitesimal rotation in a three-dimensional vector field.

∇-(del or nabla) is an operator used in vector calculus, which in terms of the rectangular Cartesian coordinates is defined as:

$$\nabla \equiv {\text{i}}\frac{\partial }{\partial x} + {\text{j}}\frac{\partial }{\partial y} + {\text{k}}\frac{\partial }{\partial z}.$$

[The representation of vector components to suitable, coordinate system (e.g., cylindrical, spherical, etc.,) relations, are independent of the coordinate system used to specify position in space and can easily be transformed].

As far as intact trees are concerned (Hejnowicz and Kurczyńska 1987; Lev-Yadun and Aloni 1990), the development of circular elements may be regarded as the manifestation of the field regions with vector rotation [curl F(*r*) ≠ 0] induced by the processes disturbing the originally present irrotational vector field [curl F(*r*) = 0]. In the case of overgrowth tissue described in this paper, at the beginning, the vector rotations [curl F(*r*) ≠ 0] are induced and are followed by the development of an irrotational field [curl F(*r*) = 0].

Considering the fact that development of circular structures is based mainly on intrusive growth, it may be hypothesized that this type of growth is induced in the region’s morphogenetic field where curl F(*r*) ≠ 0. It is important to note, that in the local regions of morphogenetic field where curl F(*r*) ≠ 0, the rotations have not always clearly manifested in cellular pattern geometry. Therefore, the small, field regions with vector rotations are likely to provide the underlying mechanism of intrusive cell growth. Such growth is exceptionally important in the differentiation of typical plant vascular systems. In this context, intrusive growth may be regarded as a mechanism eliminating (repairing) small vector rotations appearing in the morphogenetic field. Consequently, it is possible to suggest that symplastic growth, typical of plants, could be linked mostly to irrotational morphogenetic field [curl F(*r*) = 0]. Small disturbances causing local vector rotations [curl F(*r*) ≠ 0] would be compensated for by intrusive growth supported by anticlinal divisions. In case of overgrown tissue presented in this work, the differentiation of cells from a homogenous mass would be initiated in organizing centers producing vortices in regions where curl F(*r*) ≠ 0 and lead to formation of circular elements. Continued intrusive growth of newly formed cells may result in oriented axial polarization of tissue and disappearing the morphogenetic field regions with vector rotation. In this case, intrusive growth appears to provide a mechanism enabling easier transition to an ordered state.

This discussion raises a question about the nature of the hypothetical morphogenetic field in tree stem. The studies on plant growth and differentiation mechanisms indicate that the dominant role in the integration and coordination of these processes is played by cell polarity and the associated polar transport of auxin (Prasad and Dhonukshe 2013). The mechanism regulating the direction of auxin transport is primarily affected by the polar localization of the PIN auxin efflux carrier proteins (Sauer et al. 2006; Wiśniewska et al. 2006; Dhonukshe et al. 2008, 2010). The results of the present work can provide opportunities for further studies on the modifications of polarity at the cellular and subcellular level. Opportunities are also provided for explaining the effect on the geometry of supracellular morphogenetic fields and the appearance of local rotations in such fields.
